# Standardization of case definition and development of early-warning model for acute respiratory infection syndromes based on Yinzhou Regional Health Information Platform

**DOI:** 10.3389/fpubh.2025.1593102

**Published:** 2025-05-14

**Authors:** Tianfei Yi, Junfeng Zhang, Peng Shen, Yexiang Sun

**Affiliations:** Yinzhou District Center for Disease Control and Prevention, Ningbo, Zhejiang, China

**Keywords:** acute respiratory infection syndromes, influenza-like illness syndromes, epidemic criteria, historical limits method, moving percentile method, cumulative sum control chart, exponentially weighted moving average

## Abstract

**Background:**

Acute respiratory infection syndromes (ARIs) pose major public health challenges due to their high infectivity, rapid transmission, and the lack of standardized definitions balancing sensitivity and specificity in current surveillance systems.

**Objective:**

Using data from Yinzhou Regional Health Information Platform (YRHIP), we refined ARIs definition, improved classical epidemic criteria and designed a comprehensive graded early-warning model to enhance early response capabilities.

**Methods:**

We optimized ARIs definition based on laboratory-confirmed cases and evaluating screening performance with clinical diagnoses. Anomaly detection methods, including historical limits method (HLM), moving percentile method (MPM), cumulative sum control chart (CUSUM), and exponentially weighted moving average (EWMA), were employed to develop a graded early-warning model. Syndrome selection and parameter tuning were guided by Youden’s index, agreement rate and F1-score.

**Results:**

The refined ARIs definition includes: Acute-phase fever with at least one typical respiratory symptoms; or acute-phase fever with at least two atypical respiratory symptoms; or at least one typical respiratory symptoms combined with at least two atypical respiratory symptoms. Furthermore, we demonstrate that ARIs outperform ILIs definition in early screening due to their broader symptom scope. By leveraging multidimensional time series data, we developed a robust epidemic criteria framework for early-warning models. The optimal early-warning parameters included configurations of HLM (*K* = 0.8), MPM (85th percentile), CUSUM(*K* = 0.7, *H* = 5), and EWMA (*K* = 3, *λ* = 0.05). The graded early-warning system revealed: Red early-warnings (all four models triggered) had the highest specificity; Orange early-warnings (at least three models triggered) demonstrated the best overall performance; Amber early-warnings (at least two models triggered) captured subtle trends; Green early-warnings (at least one model triggered) provided the highest sensitivity.

**Conclusion:**

This study establishes an optimized, multi-model-based framework for ARIs early-warning that balances sensitivity and specificity to strengthen public health management against diverse pathogens.

## Background

The evolution of infectious disease early-warning models and syndromic surveillance systems has been fundamentally motivated by the public health necessity for timely identification and rapid response to aberrant epidemiological patterns or nascent disease trends (1). While originally conceived as countermeasures against bioterrorism threats in their embryonic stages (2), these surveillance frameworks have progressively evolved into comprehensive sentinel systems addressing multifaceted public health priorities.

The global evolution of respiratory infectious disease surveillance and early-warning architectures has undergone progressive refinement over decades, with pioneering nations including the United States, Canada, and the United Kingdom establishing robust operational frameworks. Epitomized by the US Centers for Disease Control and Prevention (US CDC) as the vanguard institution, these systems employ advanced symptom-centric surveillance frameworks exemplified by two landmark platforms: Biosurveillance Initiative for Operational Notification, Situational Awareness, and Epidemiology (BioSense) and Electronic Surveillance System for the Early Notification of Community-based Epidemics (ESSENCE). The BioSense system integrates national public health data from emergency departments, laboratories, and healthcare facilities for early disease warnings (3), while ESSENCE specializes in real-time respiratory infection monitoring through emergency department syndromic analysis (4). Parallel to North American developments, Canada’s FluWatch system demonstrates an integrated surveillance paradigm that combines virological surveillance data with population-level influenza-like illness (ILI) indicators, employing wavelet analysis for epidemic curve decomposition and threshold determination (5). China emerged as a significant contributor to digital disease surveillance through the 2004 implementation of its web-based National Notifiable Disease Reporting System (NNDRS), which established critical infrastructure for subsequent early-warning systems (6). Subsequently, the Chinese Center for Disease Control and Prevention (CHN CDC) began researching detection and early-warning technologies for infectious diseases, employing various warning methods such as fixed threshold methods, time models, and spatiotemporal models. In April 2008, a nationwide pilot of the China Infectious Diseases Automated-alert and Response System (CIDARS) was launched (7). Recently, research and medical institutions have implemented natural language processing techniques to automatically classify chief complaints and diagnostic codes in electronic health records, enabling real-time syndromic surveillance (8). While these systems have made significant advances in real-time data collection and anomaly detection, most remain limited to single-algorithm approaches, with insufficient research on integrated multi-algorithm systems or graded early warning functionality.

Given the inherent delays in diagnosing infectious diseases (9), many health organizations worldwide have adopted syndromic surveillance as a strategy for early detection and monitoring (10). This approach focuses on tracking syndromes rather than specific diseases, aiming to quickly identify clusters of unusual activity and enable timely public health interventions (11). Acute Respiratory Infection Syndromes (ARIs) refer to a spectrum of contagious respiratory disorders clinically defined by the presence of fever and/or acute respiratory symptoms, with its classification system distinguishing upper respiratory infection (URI) from lower respiratory infection (LRI) based on anatomical involvement (12). ARIs possess distinct characteristics, including diverse pathogens (13–15), high infectivity (16), rapid transmission (17), the ability to spread during the incubation period (18), and sensitivity to environmental factors (19). These characteristics pose greater challenges for control and prevention compared to other infectious diseases. Currently, no unified global standard exists for defining ARIs. This lack of standardization poses challenges for accurate early warning of ARIs and complicates disease surveillance and control efforts. Institutions including the World Health Organization (WHO) typically employ the influenza-like illness (ILI) case definition when monitoring respiratory infections potentially caused by influenza viruses or other pathogens (20). ILIs prioritize fever with cough/sore throat but overlook critical atypical symptoms (e.g., fatigue, gastrointestinal manifestations). This oversight limits their ability to detect pathogens with non-classical presentations, such as COVID-19 variants causing anosmia or pediatric RSV infections presenting with wheezing and irritability. Furthermore, in practice, the European Centre for Disease Prevention and Control (ECDC) reported low sensitivity of the method (21). Atypical symptoms, while individually non-specific, significantly enhance diagnostic precision when presented in clusters, especially in conjunction with typical symptoms. This finding, while counterintuitive, reflects the heterogeneous nature of real-world cases. The choice of a case definition plays a crucial role in determining the specificity and sensitivity of surveillance systems. Achieving an optimal balance in case definition is essential for effective ARIs early-warning.

This study is based on the Yinzhou Regional Health Information Platform (YRHIP), utilizing big data technology and natural language algorithms to refine the definition standards for ARIs. Using pathogen-positive cases and clinically diagnosed cases as the validation set, the study further optimizes the combination of symptom definition and verifies the advantages of the optimal symptom definition combination for ARIs in respiratory infectious disease surveillance, compared to the ILIs definition. The revised case definition integrates acute-phase fever with both commonly observed symptoms (such as cough and nasal congestion) and less common but clinically relevant manifestations (including fatigue, gastrointestinal discomfort, and sensory abnormalities). This expanded inclusion improves diagnostic power by capturing the broader clinical variability seen in real-world cases. Ultimately, by exploring various anomaly detection algorithms, we propose a robust, comprehensive graded early-warning model, which aims to enhance syndromic surveillance by achieving a more optimal balance between sensitivity and specificity. This system can capture potential outbreak signals promptly and enhance adaptability to complex environments, thereby alleviating the economic burden and health threats posed by the disease.

## Methods

### Study setting and population

The data for this study are derived from the YRHIP, a health information platform in eastern coastal China, covering a resident population of 1.69 million in 2023 (22). Sourced from a network of five hospitals (both public and private) and 289 primary care institutions across Yinzhou, the YRHIP comprises comprehensive electronic medical record (EMR) data, including outpatient, emergency, and inpatient visits, primary and secondary diagnoses, laboratory tests, and medication usage (23).

### Data sources

Pathogen-positive cases were obtained from *clinical laboratory report*, which aggregate data uploaded by the laboratory information system (LIS). This study utilized the thirteen respiratory pathogens published by the National Disease Control and Prevention Administration (NDCPA) as screening criteria, including: *novel coronavirus*, *influenza viruses* (*H1N1*, *H3N2*, *B-type Victoria lineage*, *B-type Yamagata lineage*, and *other subtypes*), *respiratory syncytial virus*, a*denovirus, metapneumovirus*, *rhinovirus*, *parainfluenza virus*, *common coronaviruses*, *bocavirus*, enterovirus, *Mycoplasma pneumoniae*, *Chlamydia pneumoniae*, and *Streptococcus pneumoniae* (24). Clinical diagnostic cases and syndromic cases are obtained from the *outpatient clinic daily log* and the *outpatient medical record*, respectively, with all information uploaded by the hospital information system (HIS). Clinical diagnostic cases, determined by clinical experts, were selected based on diagnostic codes for all respiratory symptom-related diseases (RSDs) according to ICD-10 classification. The specific RSDs codes are detailed in Supplementary Table S1. The WHO defines the influenza-like illness syndromes as the presence of fever (≥38°C) accompanied by a cough. Similarly, the CDC defines it as fever (≥37.8°C in the USA and ≥38°C in China) combined with either a cough or sore throat (25, 26). However, the actual symptoms often extend beyond these criteria. Considering the diverse symptomatology of acute respiratory infections, we expanded the definition of ARIs to encompass a combination of multiple symptoms, building upon existing ILIs criteria. Based on a review of existing literature and expert assessments (27–39), we categorized ARIs into three primary groups: acute phase fever symptoms, typical respiratory infection symptoms (including cough, throat discomfort, nasal symptoms, and pulmonary auscultation abnormalities), and atypical respiratory infection symptoms (encompassing fatigue, head, digestive, locomotor system, cardiopulmonary, psychiatric symptoms, sensory abnormalities, and rash symptoms). Cases presenting two or more symptom categories were classified as ARIs. The standards for natural language recognition are provided in Supplementary Table S2.

### Study design

**Figure 1:**
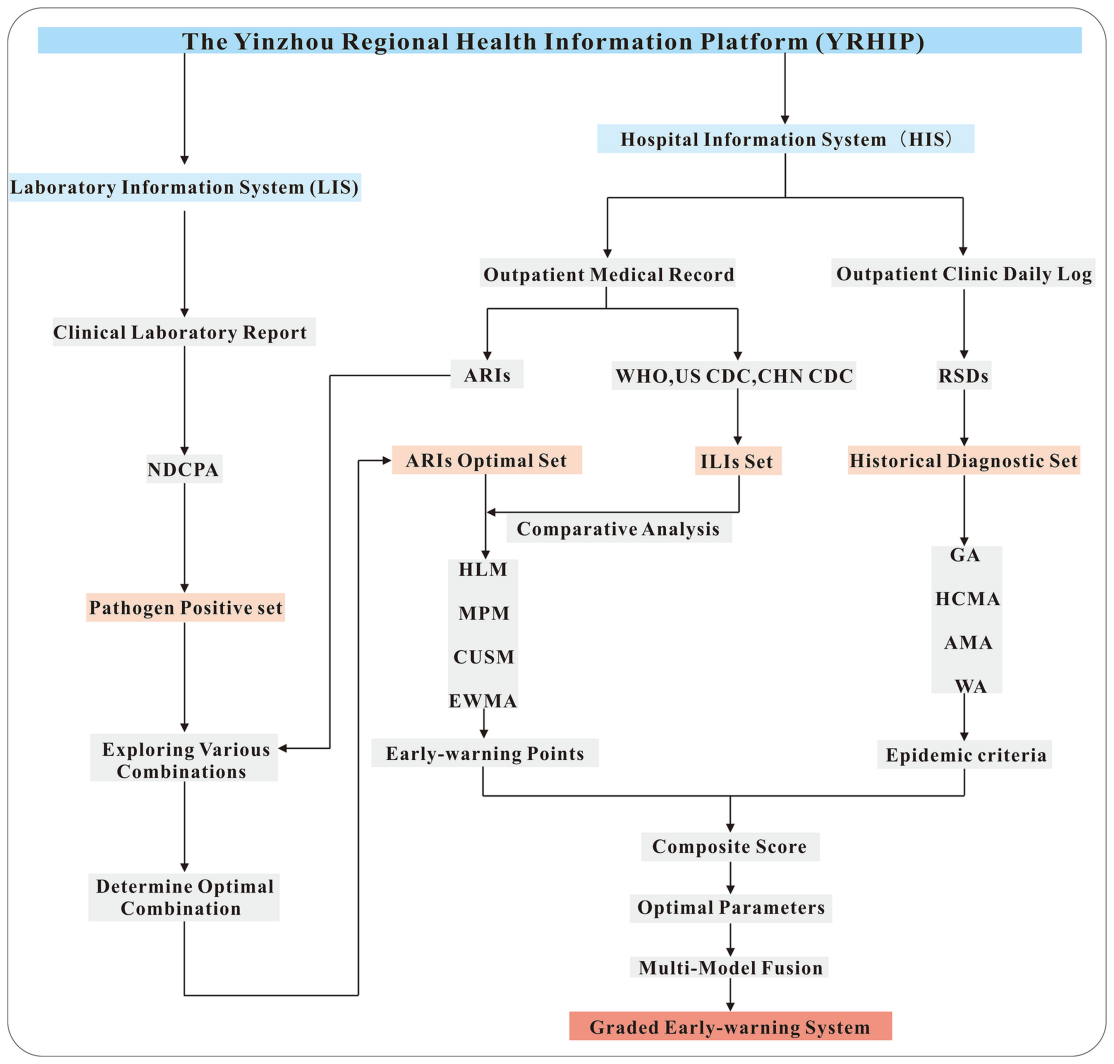
Flow chart of this study.

The workflow of this study is illustrated in Figure 1. From the LIS, laboratory-confirmed positive cases for 13 respiratory pathogens designated by the NDCPA were extracted to form a symptom validation set. Symptom profiles linked to these cases were analyzed to determine the optimal combination of symptoms for defining ARIs. Concurrently, the HIS provided a historical diagnostic set of outpatient records with ICD-10 codes for respiratory symptoms, enabling a comparative assessment of the performance of ARIs and ILIs definitions in real-time case identification. The syndrome set demonstrating optimal screening performance was subsequently applied to anomaly detection algorithms (HLM, MPM, CUSUM, EWMA). By integrating multi-temporal data (historical, current, and future information) and incorporating expert manual validation procedures, we established refined epidemic criteria to optimize model parameters. Finally, a graded early-warning system was established by employing a multi-model ensemble strategy to integrate the outputs of various predictive models. This workflow not only standardizes ARI case definitions but also establishes a robust, multi-model early-warning system capable of adapting to seasonal variability and emerging respiratory threats.

### Set up the epidemic criteria

The formula for calculating the daily diagnostic rate (DDR) of clinical diagnostic cases is as follows:


$$\mathrm{DDR}=\frac{N}{M}$$

N represents the daily diagnostic count of RSDs, while M denotes the total daily diagnostic count across all diseases. The daily diagnostic rate for syndrome cases is calculated using the same method, where N represents the daily screening count of respiratory symptom-related diseases, and M represents the total number of daily visits.

The classical epidemic definition, which describes disease incidence exceeding historical baseline levels, is qualitative in nature. To operationalize this concept, Yang et al. (40) utilized an expert consultation method to establish a quantitative threshold, defining an epidemic as occurring when incidence surpassed Y .


$$Y=\bar{X}+2S$$

$\bar{X}$ represents the historical contemporaneous moving average, S denotes the corresponding standard deviation.

Building upon the foundational work of Yang et al., this study expands the classical definition of epidemics to address its limitations in early-warning sensitivity and specificity. The specific methodology is outlined as follows.

(1)   Taking into account the impact of COVID-19 on respiratory infectious diseases, only historical diagnostic data from 2009 to 2019 were included in the calculation of the global average (GA) for clinical diagnostic cases:

$${\bar{X}}_{\mathrm{GA}}=\frac{\sum_{i=2009-1-1}^{2019-12-31}N_{i}}{\sum_{i=2009-1-1}^{2019-12-31}M_{i}}$$

(2)   Next, calculate the historical contemporaneous moving average (HCMA) and historical contemporaneous standard deviation (HCSD)of the diagnostic rate for the seven days before and after the same period in the previous five years, as well as the ambispective moving average (AMA) for the seven days before and after the specified date:

$${\bar{X}}_{\text{HCMA}}=\frac{\sum_{j=1}^{5}\sum_{i=-7}^{7}N_{y-j,d+i}}{\sum_{j=1}^{5}\sum_{i=-7}^{7}M_{y-j,d+i}}$$

$$\sigma_{\text{HCSD}}=\sqrt{\frac{\sum_{j=1}^{5}\sum_{i=-7}^{7}{\left(DR_{y-j,d+i}-{\bar{X}}_{\text{HCMA}}\right)}^{2}}{\sum_{j=1}^{5}\sum_{i=-7}^{7}M_{y-j,d+i}}}$$

$${\bar{X}}_{\mathrm{AMA}}=\frac{\sum_{-7}^{7}N_{d+i}}{\sum_{-7}^{7}M_{d+i}}$$

y represents the years (with 2009 to 2013 as the foundational dataset and 2014 to 2024 as the computational dataset), and d represents the dates (January 1 to December 31).

(3)   To simultaneously account for historical seasonality,current information and future trends, we calculated the weighted average (WA) by integrating HCMA and AMA:

$${\bar{X}}_{\mathrm{WA}}=\omega \cdot {\bar{X}}_{\text{HCMA}}+(1-\omega ){\bar{X}}_{\mathrm{AMA}}$$

ω is the weight factor, which ranges from 0 to 1. In this study, we have tentatively set it to 0.5.

(4)   Finally, we established the epidemic criteria for determining alert thresholds (2014–2024): The DDR of clinical diagnostic cases shows a significant upward trend when $\mathrm{DDR}>{\bar{X}}_{\mathrm{WA}}$ ; The DDR of clinical diagnostic cases shows a slight upward trend when $\mathrm{DDR}>{\bar{X}}_{\mathrm{WA}}$ and $\mathrm{DDR}>{\bar{X}}_{\mathrm{GA}}$ ; The DDR of clinical diagnostic cases shows a significant downward trend when $\mathrm{DDR}>{\bar{X}}_{\mathrm{WA}}$ and $\mathrm{DDR}>{\bar{X}}_{\mathrm{GA}}$ ; The DDR of clinical diagnostic cases shows a slight downward trend when $\mathrm{DDR}>{\bar{X}}_{\mathrm{WA}}$ ; Alert signals meeting criterion ${\bar{X}}_{\mathrm{WA}}<\mathrm{DDR}<{\bar{X}}_{\mathrm{GA}}$ were subjected to expert panel review, with confirmed anomalies being systematically excluded from subsequent analysis.

### Historical limits method

The Historical Limits Method (HLM) was first introduced by the Centers for Disease Control and Prevention in 1989 (41). The core concept of HLM involves using historical data from a defined baseline period to calculate the mean and standard deviation. These statistical measures are then employed to establish a range within which current observations are expected to fall. Observations falling outside this range are flagged as potential anomalies. The upper historical limit (UHL) is defined as follows:


$$\mathrm{UHL}=\sigma_{\text{HMSD}}\cdot \kappa +{\bar{X}}_{\text{HCMA}}$$

k is the confidence coefficient (one-sided).

The daily diagnostic rate is compared to the UHL. If the current value exceeds the UHL, it indicates a potential anomaly, prompting an alert for further investigation. By testing different ranges for the parameter *k*, the optimal early-warning model can be identified.

### Moving percentile method

The Moving Percentile Method (MPM) is a robust, non-parametric approach to anomaly detection that effectively identifies unusual patterns and outliers in real-time data (42, 43). It uses a moving window of historical data to calculate dynamic percentile thresholds. Considering the seasonal nature of ARIs, this study employs a 365-day moving window. Within this historical window, the data is arranged in ascending order to generate a sorted sequence:


$$W=[X_{1},X_{2},\cdots ,X_{365}]$$

The index *j* for the *p%* percentile position is calculated using the following formula:


j=p∗365

The *p%* percentile corresponds to the value at the *j-th* position in *W*. If *j* is not an integer, it is rounded up to the nearest whole number.

When the *DR* exceeds *X_j_*, it is classified as an “anomaly” and triggers a warning. By testing various ranges for the percentile *p%*, the optimal warning model can be determined.

### Cumulative sum control chart

The Cumulative Sum Control Chart (CUSUM) model enhances sensitivity in detecting small shifts in the process by cumulatively summing the deviations between actual values and reference values, thereby effectively amplifying subtle changes (44). The formula is as follows:


$$C_{t}=\max\{0,X_{t}-(\mu_{t}+\mathrm{k\sigma})+C_{t-1}\}$$

H=h∗σ

The initial value is set at C0 = 0, and kσ represents the allowable deviation. If the mean shifts from *μ_t_* to *μ_t_* + *kσ*, this triggers an alert. *H* is the decision threshold, where Ct ≥ *H* indicates a statistically significant increase. Based on their sensitivity in identifying anomalies, Ct is classified into three categories: C1 - MILD (referred to as C1), C2 - MEDIUM (referred to as C2), and C3 - ULTRA (referred to as C3). C1 has the lowest sensitivity, followed by C2, while C3 exhibits the highest sensitivity. This plan adopts a moving average period of 7 times unit, with the calculation formulas for C1, C2, and C3 as follows:


$$C_{1}=\max\{0,X_{t}-(MA_{1}+kS_{1})+C_{t-1}\}$$

$$C_{2}=\max\{0,X_{t}-(MA_{2}+kS_{2})+C_{t-1}\}$$

C3 is defined as the sum of C_t_, Ct-1 and Ct-2 derived from the C2 formula. Here, MA1 and S1 represent the moving average and moving standard deviation of reported cases from *t*-7 to *t*-1, while MA2 and S2 represent the moving average and moving standard deviation from *t*-9 to *t*-3. The parameters *h* and *k* are critical in the CUSUM model, as their values influence the model’s ability to detect anomalies. By testing different ranges for *h* and *k*, the optimal early-warning model can be established. To enhance the specificity of the CUSUM model’s predictions and minimize the risk of false alarms, this study integrates simultaneous early-warning signals from C1, C2, and C3 as alerts for ARIs.

### Exponentially weighted moving average

The Exponentially Weighted Moving Average (EWMA) model is a type of moving average that applies exponentially decreasing weights to historical data (45). This approach gives greater emphasis to recent observations while still considering older data, albeit with diminishing influence. The calculation formula is as follows:


$$E_{t}=(1-\gamma )\cdot E_{t-1}+\gamma \cdot N_{t}$$

*γ* is a model parameter, where a larger value indicates greater weight is assigned to recent data in the predictions.

The formula for calculating the upper control limit (UCL) is as follows:


$$\mathrm{UCL}=E_{t}+\kappa \cdot \sigma \cdot \sqrt{\frac{\gamma }{2-\gamma }}$$

*k* is a threshold parameter representing a specific confidence level, while *σ* denotes the standard deviation of baseline data from the past 5 years. If the current value exceeds the UCL, it indicates a potential outbreak. This study determines the optimal warning model by establishing various ranges for the parameters *γ* and k.

### Model fusion

Finally, after optimizing the parameters, the outputs of all models were integrated to establish a graded early-warning system. A red alert is issued when all four models generate warnings; an orange alert is triggered when three or more models issue warnings; a amber alert is indicated when two or more models issue warnings; a green alert is raised when at least one model issues a warning; and no alert is issued if none of the models generate warnings.

### Statistical analysis

#### Parameter range determination

To determine the candidate parameter ranges for each model, we adopted an empirical tuning strategy informed by three factors: (1) previous literature on infectious disease surveillance and statistical control charts, such as EWMA (46), (2) expert consensus among epidemiologists and informatics specialists at the Yinzhou CDC, such as MPM, and (3) preliminary exploratory data analysis conducted on historical respiratory infection datasets from the YRHIP platform, such as HLM and CUSUM. This hybrid approach ensures that the final parameter space was not arbitrarily defined, but rather grounded in evidence, expert interpretation, and data-driven validation, thereby improving the transparency and reproducibility of the early-warning system.

#### Screening performance evaluation

To evaluate the performance of various parameters, we utilized a comprehensive scoring method that integrates the Youden’s index, F1-score, and agreement rate.


$$\text{Youden}\prime s\;\text{index}=\frac{A}{A+C}+\frac{D}{B+D}-1$$

$$F1\;\text{score}=\frac{2A}{2A+B+C}$$

$$\text{Agreement rate}=\frac{A+D}{A+B+C+D}$$

A represents true early-warning, B represents false early-warning, C represents false non-early-warning, D represents true non-early-warning.

Each metric was standardized to ensure comparability:


$$M=\frac{M-\min(M)}{\max(M)-\min(M)}$$

The standardized values were then aggregated to calculate an overall composite score:


$$\text{Composite score}=M_{\text{Youden}\prime s\;\text{index}}+M_{F1\;\text{score}}+M_{\text{Agreement rate}}$$

This method provides a balanced assessment of model performance across multiple dimensions.

#### Mean squared error

We used the Mean Squared Error (MSE) to quantify the discrepancy between ARIs/ILIs and clinically diagnosed cases. The calculation formula is as follows:


$$\mathrm{MSE}=\frac{1}{n}\sum_{i=1}^{n}{\left(\overset{⌢}{y}i-\mathrm{yi}\right)}^{2}$$

#### Multi-group difference analysis

To evaluate overall differences among multiple groups, a two-way ANOVA was conducted. *Post hoc* pairwise comparisons were then performed using the Bonferroni correction method to account for multiple testing.

## Results

### Optimizing ARIs criteria

**Figure 2:**
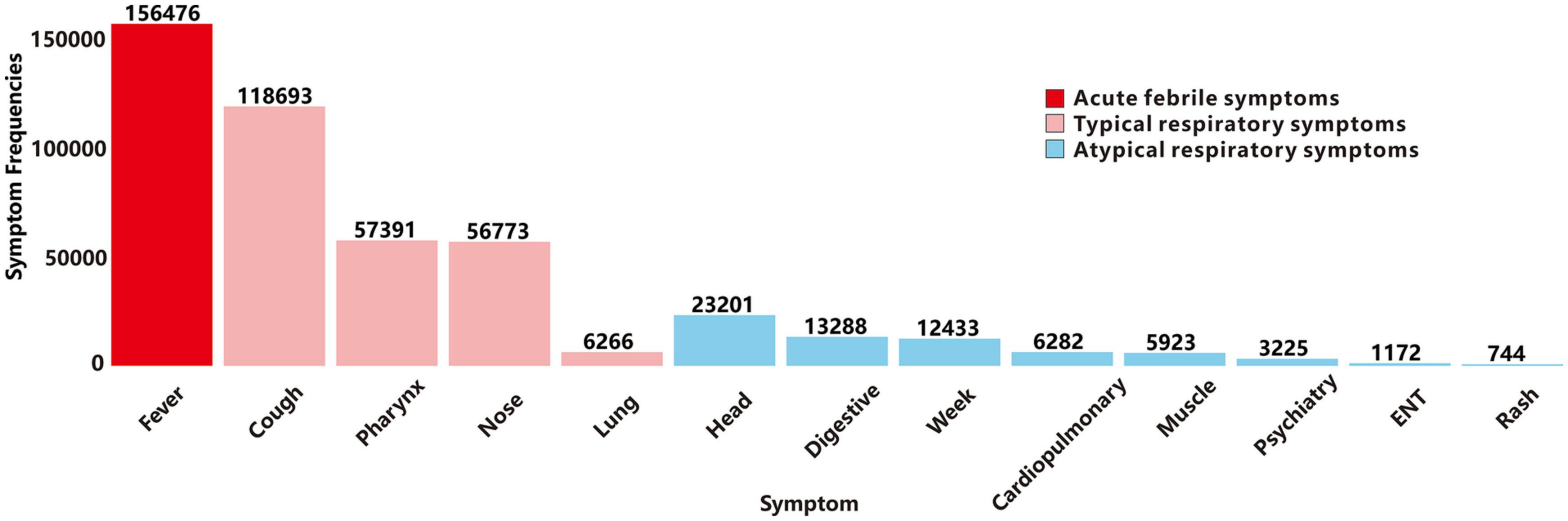
Frequency distribution of the main symptoms of ARIs.

Based on YRHIP system data from 2019 to 2024, the *clinical laboratory report* recorded a total of 62,487,623 laboratory test records. Among these, 10,807,158 tests screened for thirteen target respiratory pathogens, identifying 424,234 positive cases and 10,382,924 negative results. Subsequently, we analyzed the frequency of the three primary symptom categories of ARIs in cases testing positive for thirteen respiratory pathogens (Figure 2). Acute-phase fever showed the highest prevalence, followed by typical respiratory infection symptoms. An exception was abnormal lung auscultation, which, while less commonly observed, demonstrated high specificity for respiratory diseases. Atypical symptoms were the least frequently reported among positive cases.

**Figure 3:**
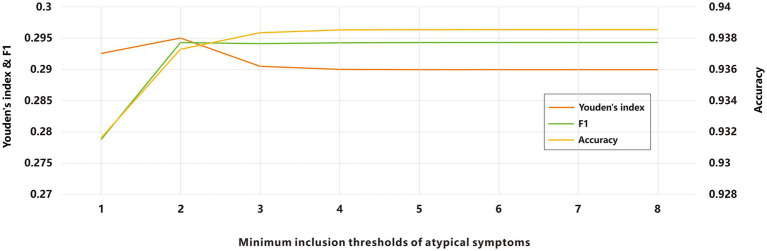
Evaluation of ARIs screening based on different minimum inclusion thresholds of atypical symptoms.

Due to the prevalence of atypical symptoms, relying solely on a single-symptom screening for ARIs screening could increase the misdiagnostic rate, while incorporating all symptoms might elevate missed-diagnosis rates. To optimize this balance, we investigated the minimum inclusion threshold for atypical symptoms. Using a panel of thirteen pathogens as the symptom validation set, we evaluated various combinations of atypical symptoms across different minimum inclusion thresholds as screening criteria. The results demonstrated that as the minimum inclusion threshold for atypical symptoms increased, the agreement rate improved (Figure 3). Subsequently, we calculated the Youden’s index and F1-score, both of which peaked when the minimum inclusion threshold was set to 2. To identify the optimal cut-off point, we computed a composite score, which also reached its maximum when the minimum inclusion threshold was set to 2. Based on these findings and the definition of ILIs, we propose the following final criteria for defining ARIs: acute-phase fever symptoms combined with at least one typical respiratory infection symptoms; or acute-phase fever symptoms combined with at least two atypical respiratory infection symptoms; or at least one typical respiratory infection symptoms combined with at least two atypical symptoms.

### Screening evaluation of ARIs and ILIs definition

According to the YRHIP data, from 2009 to 2024, the *outpatient clinic daily log* recorded a total of 184,961,667 clinical diagnoses, including 27,935,373 cases classified under ICD codes related to RSDs. Since patients assigned ICD codes were diagnosed by clinical experts, these diagnoses are considered highly authoritative. Consequently, this cohort was used for subsequent model validation. Additionally, from 2019 to 2024, the *outpatient medical record* documented 39,859,297 outpatient visits, of which 1,340,626 met the ARIs criteria. In comparison, applying the ILIs definition from the WHO, US CDC, and CHN CDC identified 977,753, 1,156,905, and 1,154,118 cases, respectively.

**Figure 4:**
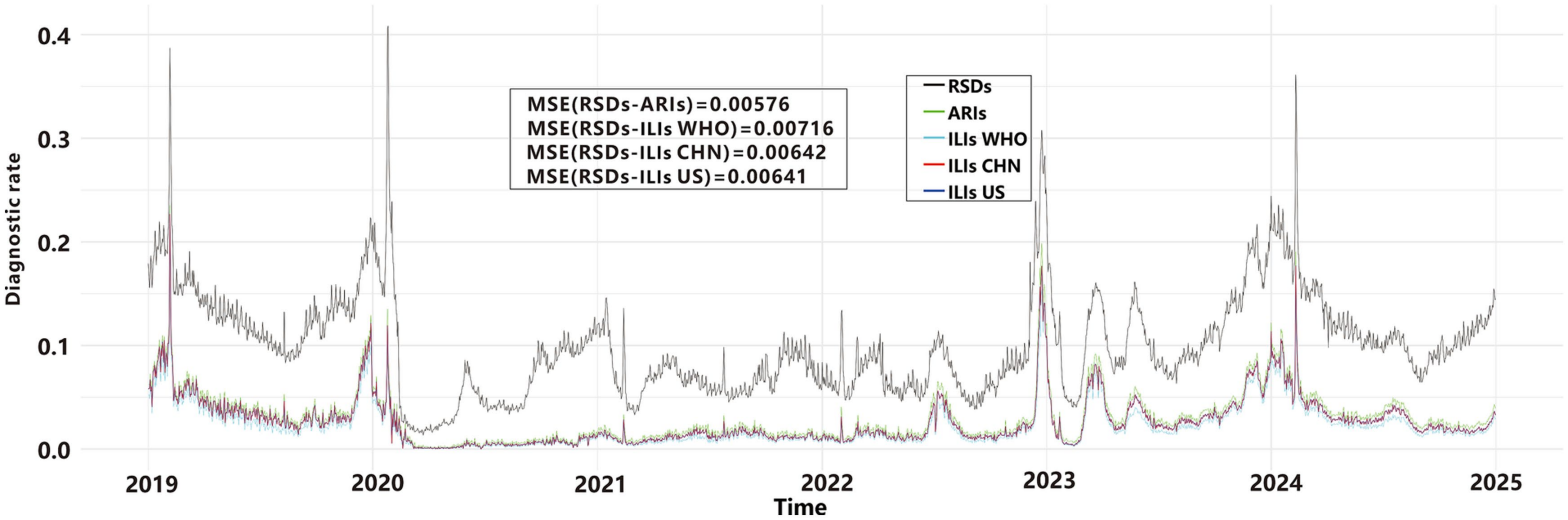
Daily diagnostic rates for ARIs, ILIs, and RSDs.

We used line graphs to depict the daily diagnostic rate of ARIs, ILIs, and RSDs. A two-way ANOVA (Table 1) revealed significant differences between syndromic surveillance (ARIs and ILIs) and clinical diagnoses after accounting for the blocking effect of Date (*p* < 0.001), To determine which syndrome exhibited the smallest discrepancy from actual clinical cases, we performed a MSE analysis. The results revealed that ARIs and RSDs had smaller discrepancies in DDR compared to ILIs (Figure 4). Using RSDs as diagnostic validation set and applying the ARIs and ILIs definition as screening tests, we evaluated the screening performance of each definition. Table 2 presents a comparative analysis of diagnostic metrics, including Youden’s index, accuracy, and F1-score, for various ARIs and ILIs definition. A two-way ANOVA revealed significant differences in Youden’s index, accuracy rate and F1-score (*p* < 0.001) after accounting for the blocking effect of Date. *Post hoc* Bonferroni analysis further indicated that the screening value of the ARIs definition surpasses that of the ILIs definition.

**Table 1:** Two-way ANOVA of DDR for ARIs and ILIs definitions.

Indicators	Group	Date	RSDs-ARIs	RSDs-WHO	RSDs-US	RSDs-CHN
*F*-value	12445.314	19.846	Bonferroni	Bonferroni	Bonferroni	Bonferroni
*p*-value	<0.001	<0.001	<0.001	<0.001	<0.001	<0.001

**Table 2:** Two-way ANOVA of diagnostic value for ARIs and ILIs definitions.

Indicators	$\bar{X}\pm S$				Group		Bonferroni *P*-value		
	ARIs	WHO	US	CHN	*F*-value	*P*-value	ARIs-WHO	ARIs-US	ARIs-CHN
Youden’s index	0.187 ± 0.086	0.136 ± 0.073	0.168 ± 0.082	0.168 ± 0.083	9226.318	<0.001	<0.001	<0.001	<0.001
Accuracy rate	0.907 ± 0.042	0.904 ± 0.044	0.906 ± 0.043	0.906 ± 0.043	1804.193	<0.001	<0.001	0.220	0.035
F1-score	0.299 ± 0.117	0.232 ± 0.109	0.275 ± 0.117	0.275 ± 0.117	11753.723	<0.001	<0.001	<0.001	<0.001

### Construction of the baseline early warning model for ARIs

**Figure 5:**
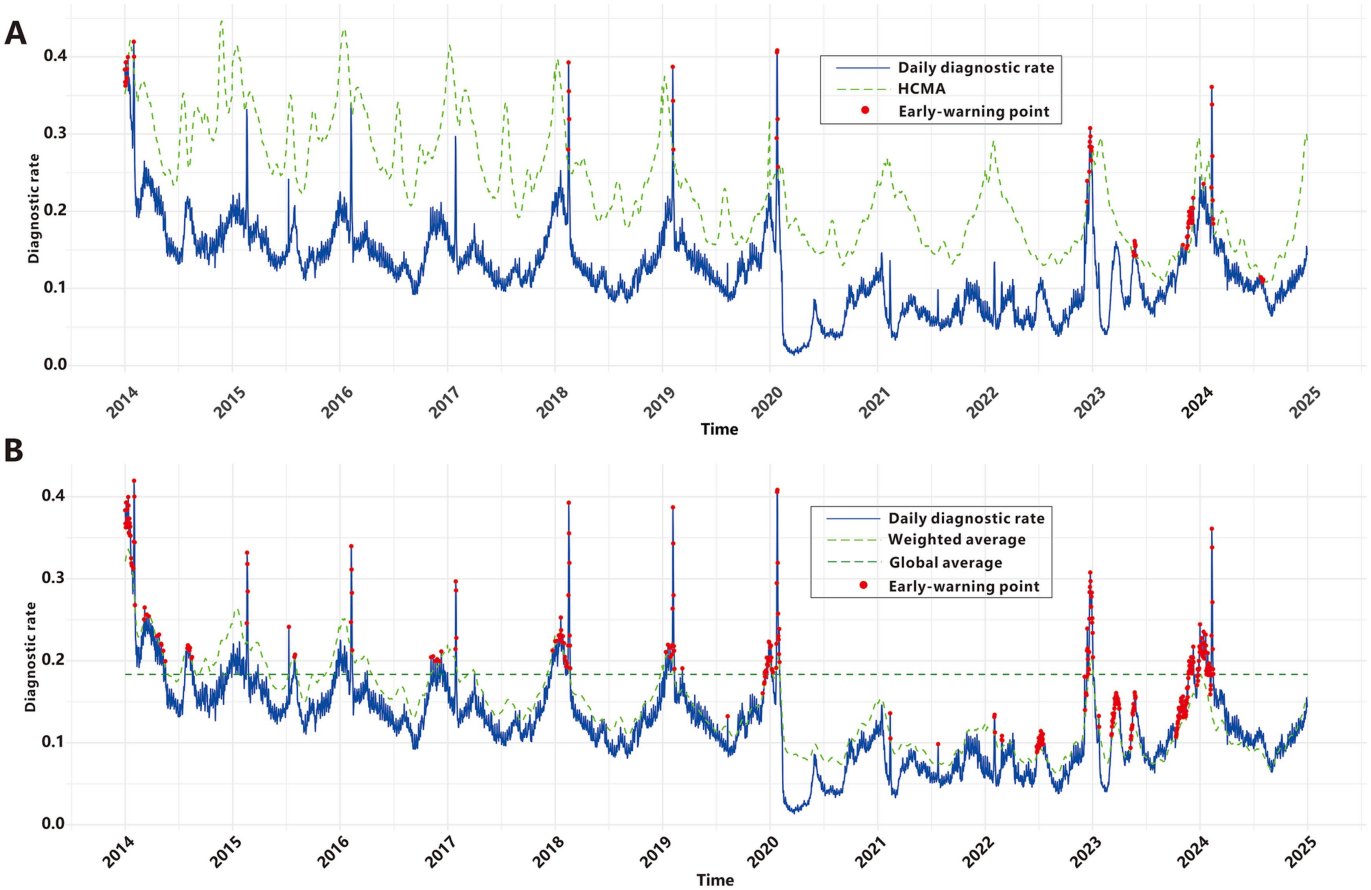
Epidemic signals based on RSDs. **(A)** Classical criteria. **(B)** Improved criteria.

Yang et al. employed the classical threshold formula to define epidemic criteria; however, our analysis revealed limitations in its real-world early-warning utility. As shown in Figure 5A, this method failed to generate any epidemic signals during the COVID-19 pandemic or the winter RSDs epidemic seasons from 2015 to 2017. Paradoxically, it produced a cluster of alerts in mid-2024 when RSDs incidence exhibited a clear downward trend. These findings underscore the necessity to develop refined epidemic criteria that balance sensitivity, specificity, and robustness. To address this, we developed new epidemic criteria based on a weighted average of multidimensional time series data—incorporating historical, current, and future trends—combined with manual validation, as detailed in the Methods section. From 2009 to 2019, the baseline RSDs diagnostic rate averaged 0.183. Using data from the first 5 years of the cohort as a reference, a total of 371 epidemic signals were generated between 2014 and 2024, with 276 signals(74.4%) occurring between 2019 and 2024 (Figure 5B). Prior to February 2020, signals predominantly occurred during winter months, precisely capturing seasonal epidemic peaks. Between 2020 and 2022, despite the impact of the COVID-19 pandemic and the absence of distinct seasonal patterns due to generally low incidence rates, the model still sensitively identified sub-epidemic spikes that exceeded epidemic criteria, generating 51 epidemic signals. Subsequently, a significant concentration of signals emerged around 2023, including 27 around the New Year period, 28 in March, 18 in May, and a cluster of 117 signals from November through the following February. From March through the end of 2024, no signals were triggered as RSDs activity remained below epidemic criteria.

Subsequently, we investigated early-warning models applicable to ARIs and identified the optimal parameters for each model (Supplementary Table S3, Supplementary Figure S1). Detailed methodological descriptions are provided in the Methods section. The parameter *K* for the HLM was examined within the range of 0.1 to 2. Based on the evaluation metrics (Supplementary Figure S2A), the model demonstrated optimal early-warning performance with a total of 402 warning points when *K* was set to 0.8, achieving a composite score of 2.80. The MPM evaluates whether a monitoring point should issue an early-warning signal by defining threshold values at different percentiles of historical data. Analysis of model performance across various percentiles revealed that the predictive performance was optimal at the 85th percentile, with a total of 288 warning points (Supplementary Figure S2B). For the CUSUM model, the parameters *K* and *H* were explored within the ranges of 0 to 1 and 0 to 10, respectively. The model achieved optimal performance when *K* = 0.7 and *H* = 5, identifying a total of 336 warning points with a composite score of 2.89 (Supplementary Figure S2C). Similarly, for the EWMA model, the optimal performance was observed with *K* = 3 and *λ* = 0.05, resulting in 314 warning points and a composite score of 2.87 (Supplementary Figure S2D).

### Graded warning system based on multi-model fusion

**Figure 6:**
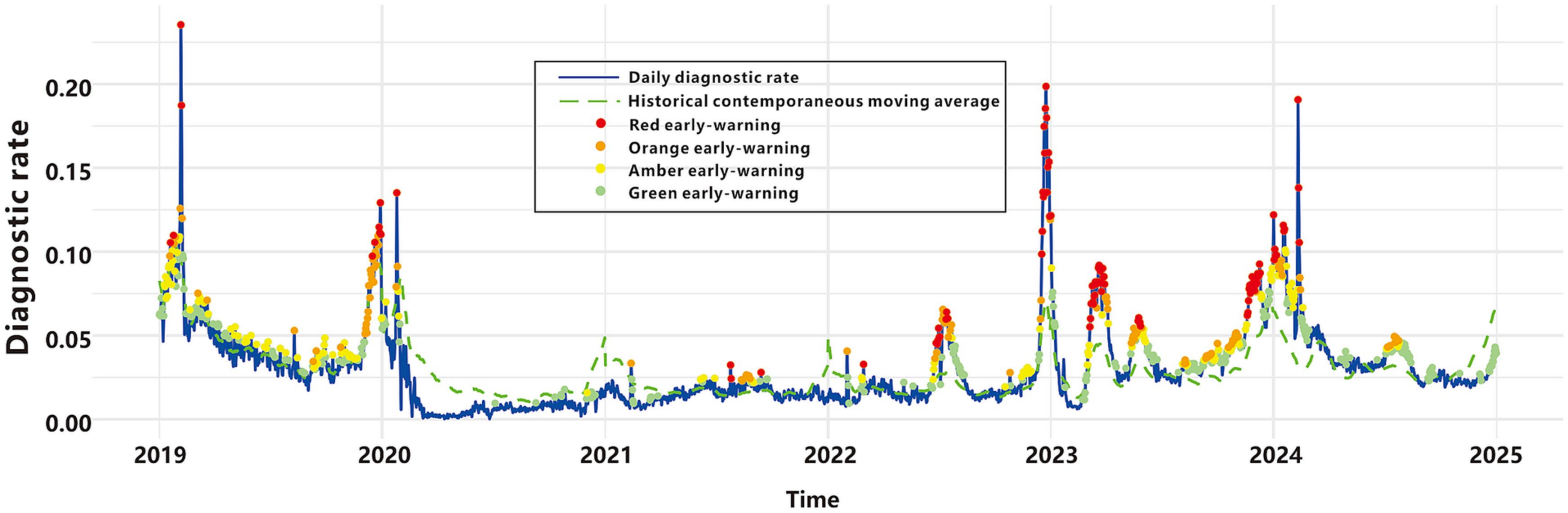
Graded early-warning system.

We integrated the outputs of four early-warning models in a parallel configuration to construct a comprehensive graded early-warning system. This system classifies warnings into four hierarchical levels based on the consistency of the models’ outputs (Table 3, Figure 6): “Red early-warnings,” represented by red points, these are triggered when all four models concur, resulting in 100 warning points. Red warnings demonstrate the highest specificity (0.997), minimizing false alarms, and show the greatest overlap with the epidemic criteria. These warnings are predominantly observed during peak epidemic periods, highlighting their accuracy and relevance in critical situations; “Orange early-warnings,” marked as orange points, these are activated when at least three models issue alerts, producing 214 warning points. This level achieves the most balanced performance, with the highest Youden’s index (0.556), F1-score (0.657), and agreement rate (0.923). Orange early-warnings appear frequently during both moderate and high-risk periods; “Amber early-warnings,” depicted by amber points, these are generated when at least two models agree, accounting for 372 warning points. Amber early-warnings are observed in intermediate risk periods, effectively capturing more nuanced trends in disease incidence; “Green early-warnings,” shown as green points, these are issued when at least one model signals an alert, leading to 654 warning points. Green early-warnings demonstrate the highest sensitivity (0.902) but the lowest specificity (0.788) and are broadly distributed across the entire timeline. This level reflects the system’s ability to capture early signals of potential risk.

**Table 3:** Performance metrics across graded early-warning system.

Warning levels	Sensitivity	Specificity	Youden’s index	F1-score	Agreement rate	Composite score
Green	0.902	0.788	0.691	0.535	0.803	1.199
Amber	0.739	0.912	0.651	0.630	0.890	2.434
Orange	0.583	0.972	0.556	0.657	0.923	2.613
Red	0.344	0.997	0.342	0.505	0.915	0.932

## Discussion

Acute respiratory infection syndromes pose a significant public health concern, necessitating robust screening criteria and early-warning tools to mitigate their clinical and epidemiological burdens. This study developed and validated a comprehensive framework for optimizing ARIs definition, evaluating screening performance, improving the classical epidemic criteria and constructing an early-warning model tailored to regional healthcare data. By leveraging an extensive datasets and integrating advanced analytical approaches, this work highlights key strategies to enhance syndrome-based surveillance systems.

Extensive literature has consistently demonstrated that syndromic cases exhibit higher timeliness compared to clinically diagnosed and pathogen-confirmed cases, though with lower specificity (10, 47–49). The refined syndrome screening criteria proposed in this study address a long-standing challenge in syndromic surveillance: the sensitivity-specificity trade-off (50). The findings demonstrate that the integration of acute-phase fever with both typical and atypical respiratory symptoms enhances diagnostic robustness. While acute-phase fever (51) and typical respiratory symptoms (52), as hallmark indicators of respiratory infections, have been consistently associated with high sensitivity, the strategic addition of atypical symptoms significantly improves accuracy—particularly when multiple symptoms co-occur. Notably, atypical symptoms’ inclusion of two or more significantly enhances diagnostic accuracy. Even when compared to the ILIs, the ARIs consistently shows superior screening performance and alignment with confirmed clinical diagnoses. A plausible explanation for this disparity lies in the greater adaptability of the ARIs to diverse clinical settings, as it encompasses a broader symptom spectrum, including both typical and atypical respiratory manifestations that traditional ILIs definition fail to capture adequately. This approach underscores the importance of flexible symptom inclusion to account for pathogen diversity and clinical variability, laying the groundwork for the development of definition for other complex disease syndromes.

While the classical epidemic criteria proposed by Yang et al. (40) provided foundational epidemiological insights, they failed to detect most pandemic signals and issued paradoxical alerts during epidemiologically quiescent periods. To address these limitations, we improved the classical epidemic criteria to capture both expected seasonal peaks and unexpected sub-epidemic spikes. Prior to 2020, epidemic signals showed distinct winter seasonality, consistent with established patterns of respiratory infection transmission (53). However, these patterns were disrupted during the COVID-19 pandemic, likely due to behavioral adaptations and policy interventions (54, 55). This disruption highlights the need to incorporate external factors, such as mask mandates and social distancing, into future prediction models. Following the gradual relaxation of non-pharmaceutical interventions (NPIs) and epidemic prevention policies, multiple infection peaks emerged in Yinzhou District since late 2022. This phenomenon, described as “immunological debt” in academic literature (56), underscores the complex dynamics of infectious disease transmission in the post-intervention era. The absence of signal disorder before and after the COVID-19 pandemic demonstrates the robustness of our criteria in capturing complex disease transmission dynamics, even under unprecedented epidemiological perturbations. Crucially, as RSDs activity remained low in mid-2024, no epidemic signals were generated. In contrast to the false signal produced by the conventional criteria during the same period, this demonstrates higher specificity. Our epidemic criteria relies on multi-temporal data (historical, current, and future) and expert validation processes, posing challenges to its real-time implementation in real-world scenarios. Nevertheless, the system’s resilient design maintains reliability during major disruptions like the COVID-19 pandemic, establishing it as a robust benchmark for early-warning model evaluation.

The global COVID-19 pandemic and subsequent emergence of multiple pathogens following NPIs relaxation emphasize the urgent need for effective ARIs early-warning systems. Timely and accurate early-warning signals enable health professionals to implement interventions during outbreak initiation (57), enable health professionals to implement interventions during outbreak initiation. Previous studies underscore the necessity of multi-model fusion to address the heterogeneity inherent in epidemiological data and the complexities of outbreak dynamics (58). Our study advances this field through a novel multi-model system that incorporates HLM, MPM, CUSUM, and EWMA methods into a graded alert framework. The graded early-warning system developed in this study harnesses the strengths of four distinct methods, generating nuanced alerts—classified as red, orange, amber, or green—based on multi-model consensus. This approach achieves a balance between sensitivity and specificity, effectively reducing the risks of false positives and false negatives. Red alerts, triggered when all four algorithms detect anomalies, may correspond to the highest level of public health intervention, including enhanced hospital preparedness, targeted community interventions and mobilized emergency task force. Orange alerts, which offer the best balance between sensitivity and specificity, may prompt increased clinical surveillance, reinforcement of infection control measures, issuance of public health advisories, or mobilization of additional healthcare personnel. Amber alerts can serve as an early caution, triggering internal reviews of case trends and cross-departmental coordination without full-scale intervention. Green alerts, while having the highest sensitivity, can support routine surveillance and background monitoring, signaling areas for focused data validation or community-level health messaging. To support real-world deployment, the alert levels could be visualized within existing digital dashboards of regional health platforms. Standard operating procedures (SOPs) linked to each alert level would ensure that the system is not only data-driven but also actionable, enabling local health authorities to scale their responses in a timely, proportionate, and resource-efficient manner. By integrating multi-model outputs, this system establishes a robust surveillance framework for respiratory infectious diseases, delivering reliable graded alerts through comprehensive analysis of complex epidemiological data.

## Conclusion

In summary, this study establishes a robust foundation for enhancing ARIs surveillance and early-warning systems. Through the optimization of ARIs definition, evaluation of screening performance, improvement of classical epidemic criteria and integration of multi-model predictions, we have developed a scalable and adaptive framework for public health management. These findings hold significant implications for the management of respiratory infectious diseases, particularly in the face of increasing pathogen diversity and evolving global health challenges.

## Data Availability

The data analyzed in this study is subject to the following licenses/restrictions: The data that support the findings of this study are available from YRHIP but restrictions apply to the availability of these data, which were used under license for the current study, and so are not publicly available. Data are however available from the authors upon reasonable request and with permission of YRHIP. Requests to access these datasets should be directed to 445934456@qq.com.

## Supplementary material

SUPPLEMENTARY FIGURE S1 Exploration of model parameters. **(A)** HLM. **(B)** MPM. **(C)** CUSUM. **(D)** EWMA.

SUPPLEMENTARY FIGURE S2 Construction of the optimal model. **(A)** HLM. **(B)** MPM. **(C)** CUSUM. **(D)** EWMA.

SUPPLEMENTARY TABLE S1 Diagnostic codes for all RSDs.

SUPPLEMENTARY TABLE S2 Natural language recognition standards for ARIs.

SUPPLEMENTARY TABLE S3 Exploration of model parameters.

## Glossary

ARIs: acute respiratory infection syndromes
YRHIP: Yinzhou Regional Health Information Platform
HLM: historical limits method
MPM: moving percentile method
CUSUM: cumulative sum control chart
EWMA: exponentially weighted moving average
US CDC: US Centers for Disease Control and Prevention
ILI: influenza-like illness
CHN CDC: Chinese Center for Disease Control and Prevention
CIDARS: China Infectious Diseases Automated-alert and Response System
URI: upper respiratory infection
LRI: lower respiratory infection
ECDC: European Centre for Disease Prevention and Control
EMR: electronic medical records
NDCPA: National Disease Control and Prevention Administration
HIS: hospital information system
LIS: laboratory information system
RSDs: respiratory symptom-related diseases
ILIs: influenza-Like illness syndromes
DDR: daily diagnostic rate
GA: global average
HCMA: historical contemporaneous moving average
HCSD: historical contemporaneous standard deviation
AMA: ambispective moving average
WA: weighted average
UHL: upper historical limit
UCL: upper control limit
MSE: mean squared error
NPIs: non-pharmaceutical interventions
NNDRS: National Notifiable Disease Reporting System
BioSense: Biosurveillance Initiative for Operational Notification, Situational Awareness, and Epidemiology
ESSENCE: Electronic Surveillance System for the Early Notification of Community-based Epidemics
