# Supplementary figures and images for: Standardization of case definition and development of early-warning model for acute respiratory infection syndromes based on Yinzhou Regional Health Information Platform
Source: Front Public Health. 2025 May 14;13:1593102. doi: 10.3389/fpubh.2025.1593102 (PMC12116594; doi:10.3389/fpubh.2025.1593102)

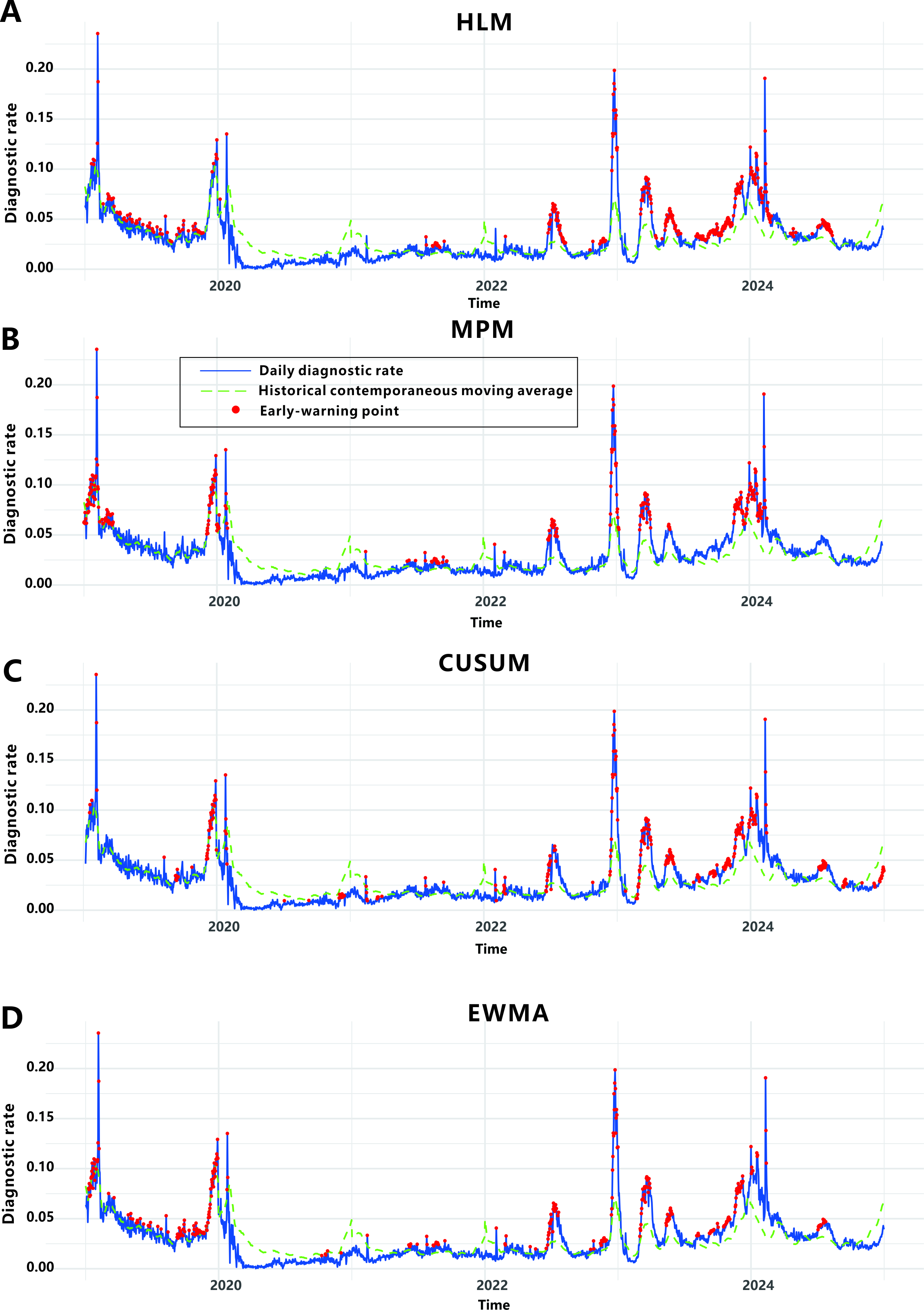

Supplement: SUPPLEMENTARY FIGURE S1 — Exploration of model parameters. (A) HLM. (B) MPM. (C) CUSUM. (D) EWMA. [file Data_Sheet_1.ZIP › Figure S2.jpg]

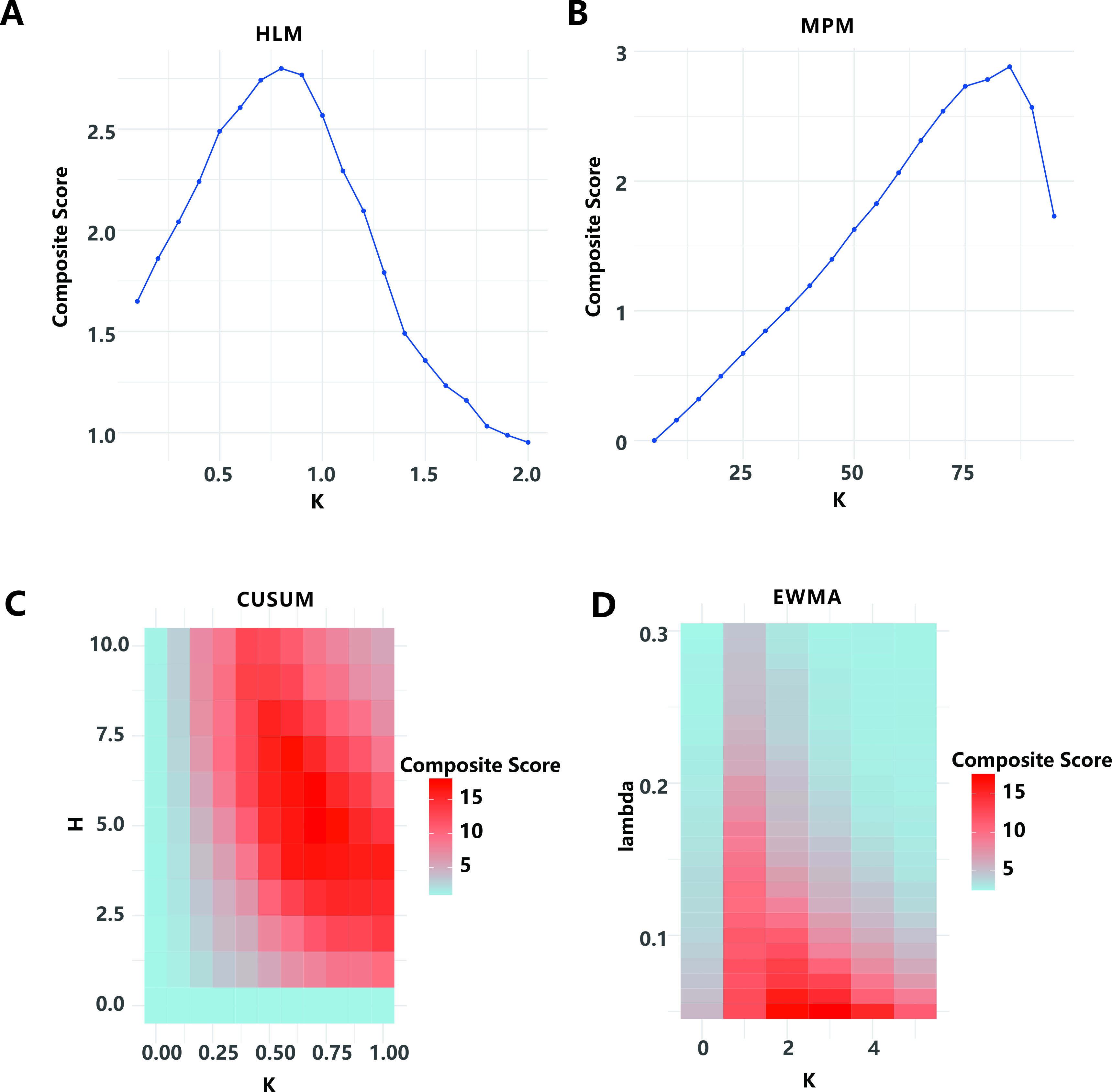

Supplement: SUPPLEMENTARY FIGURE S1 — Exploration of model parameters. (A) HLM. (B) MPM. (C) CUSUM. (D) EWMA. [file Data_Sheet_1.ZIP › Figure S1.jpg]
